# The neutrophil-to-lymphocyte ratio and platelet-to-lymphocyte ratio in patients with neuromyelitis optica spectrum disorders and multiple sclerosis

**DOI:** 10.3389/fneur.2025.1598885

**Published:** 2025-10-23

**Authors:** Qingli Sun, Xinran Ma, Linjing Zhang, Danyang Tian

**Affiliations:** Department of Neurology, Peking University Third Hospital, Beijing, China

**Keywords:** neuromyelitis optica spectrum disorders, multiple sclerosis, neutrophil-to-lymphocyte ratio, platelet-to-lymphocyte ratio, auxiliary tool

## Abstract

**Background:**

This study aimed to investigate the differences in neutrophil-to-lymphocyte ratio (NLR) and platelet-to-lymphocyte ratio (PLR) between patients with neuromyelitis optica spectrum disorders (NMOSDs) and multiple sclerosis (MS), as well as their potential associations with disease onset and progression.

**Methods:**

Clinical, laboratory, and imaging data of NMOSD and MS patients admitted to Peking University Third Hospital were retrospectively analyzed. Blood test results within 1 week of new clinical symptoms or imaging abnormalities were used to calculate NLR and PLR. These ratios were compared with those of 100 healthy controls.

**Results:**

A total of 79 NMOSD patients, 75 MS patients, and 100 healthy controls were included. The mean age of NMOSD patients was significantly higher than that of MS patients (*p* = 0.012). The Expanded Disability Status Scale (EDSS) scores at onset and after 1 year were significantly higher in NMOSD patients compared to MS patients (both *p* = 0.002). NLR was significantly elevated in NMOSD patients compared to both MS patients and healthy controls (*p* = 0.002 and *p* = 0.001, respectively), while no significant difference was observed between MS patients and healthy controls (*p* = 0.407). No significant differences in PLR were found among the three groups. After adjusting for age and gender, significant differences in NLR but not PLR remained between NMOSD and MS patients (*p* = 0.010 and *p* = 0.364). Receiver operating characteristic (ROC) analysis revealed an area under the curve (AUC) of 0.717 for NLR (*p* = 0.001, 95% CI: 0.636–0.798) and 0.567 for PLR (*p* = 0.152, 95% CI: 0.476–0.658) in distinguishing NMOSD from MS. In NMOSD patients, baseline and 12-month EDSS scores were significantly lower in the low NLR group (NLR < 2.44) compared to the high NLR group (NLR ≥ 2.44; both *p* = 0.008). Similarly, in MS patients, baseline and 12-month EDSS scores were significantly lower in the low NLR group (NLR < 1.68) compared to the high NLR group (NLR ≥ 1.68; both *p* = 0.003).

**Conclusion:**

NLR may serve as a useful auxiliary tool for differentiating acute attacks or relapses of NMOSD from MS and is associated with prognosis in both NMOSD and MS patients.

## Introduction

Neuromyelitis optica spectrum disorders (NMOSDs) and multiple sclerosis (MS) are immune-mediated demyelinating diseases of the central nervous system (CNS). Both primarily affect the optic nerve and spinal cord but can also involve other regions such as the brain, cerebellum, and brainstem. Whether NMOSD patients or MS patients, the majority of them have experienced relapses (1, 2). Before the discovery of aquaporin-4 (AQP4) antibodies, NMOSD was often regarded as a subtype of MS. However, with advancements in understanding these conditions, it is now widely recognized that NMOSD and MS are distinct entities. NMOSD is often associated with AQP4 antibodies, tends to have more severe attacks, and is less likely to show brain lesions typical of MS on MRI. In contrast, MS is characterized by oligoclonal bands in the cerebrospinal fluid, a relapsing–remitting course, and a different response to immunomodulatory therapies (1, 2). Early differentiation is critical due to significant differences in disease onset, clinical manifestations, prognosis, and treatment strategies. Currently, several examinations, including blood-based biomarkers, advanced imaging techniques, and novel immunological assays, are available to assist in differentiating NMOSD from MS (3). While lumbar puncture and MRI imaging are essential for diagnosis and differentiation, lumbar puncture is invasive and intolerable for some patients, and repeated MRI scans are costly. Therefore, there is a pressing clinical need to explore alternative diagnostic tools.

Routine blood tests, including white blood cell counts, neutrophils, and platelets, are commonly used in clinical practice to reflect systemic inflammatory changes. Increased neutrophil counts correlate with inflammatory responses triggered by MS relapses (4).

The neutrophil-to-lymphocyte ratio (NLR) reflects systemic inflammation, which is increasingly recognized as a contributor to both NMOSD and MS pathogenesis and may correlate with disease activity or prognosis. NLR serves as a biomarker for various diseases, such as myocardial infarction (5) and chronic obstructive pulmonary disease (6), and is associated with disease prognosis. Studies have shown that NLR is significantly elevated in MS patients (7) and correlates with disease activity in relapsing–remitting MS (8, 9). As an autoimmune disease, NMOSD has been less studied in relation to NLR (10). Research indicates that NLR can help differentiate NMOSD from MS and myelin oligodendrocyte glycoprotein antibody-associated diseases (MOGAD) (11, 12). Elevated NLR is an independent risk factor for neurological dysfunction severity at the onset of first-episode NMOSD (12, 13). Meta-analysis demonstrated that the NLR was significantly elevated in patients with NMOSD, and a moderate-to-low correlation existed between NLR and poor prognosis in NMOSD patients (14). Additionally, NLR could predict the Extended Disability Status Scale (EDSS) score at 6 months in pediatric NMOSD cases (15). The platelet-to-lymphocyte ratio (PLR) has also been associated with several autoimmune diseases (16, 17). Studies indicate that an increased PLR during NMOSD can predict the severity of neurological disability within 2 years in NMOSD patients. Furthermore, PLR may help differentiate MS from NMOSD (18, 19), and other studies have shown its association with MOG antibody-associated disease (MOGAD) recurrence (11). Combining NLR and PLR provides a more comprehensive reflection of systemic inflammatory changes.

This study aims to investigate differences in blood indicators such as NLR and PLR between MS and NMOSD patients, assess their potential utility as adjuncts for distinguishing these two conditions, and examine the association between these markers and patient prognosis 1 year after measurement.

## Methods

### Study population

We retrospectively analyzed data from NMOSD and MS patients admitted to Peking University Third Hospital. Inclusion criteria were: (1) NMOSD patients meeting the 2015 International NMO Diagnostic Panel criteria (1) and MS patients fulfilling the 2017 McDonald criteria (2); (2) patients who had not received glucocorticoids within 1 month prior to onset or relapse, nor used immunosuppressants or disease-modifying therapies (DMTs) within 3 months before these events; and (3) patients with complete clinical records. Exclusion criteria included: (1) severe hepatic or renal dysfunction; (2) malignancy, trauma, or other autoimmune diseases; (3) recent fever or infection; and (4) pregnancy.

### Clinical data

Clinical data comprised sex, age, disease duration, number of relapses, and EDSS scores. EDSS scores were recorded at onset/relapse and again 12 months later. Laboratory tests included complete blood counts, urinalysis, stool tests, hepatic and renal function assessments, erythrocyte sedimentation rate, thyroid function and antibodies, aquaporin 4 (AQP4) status, tumor markers, and immune-related parameters such as anti-nuclear antibodies (ANAs) and anti-extractable nuclear antigen antibodies (ENAs). All patients underwent testing for serum MOG antibodies to rule out MOGAD. AQP4 and MOG antibody detection were performed using cell-based assay (CBA). Blood test results obtained within 1 week of new clinical symptoms or imaging findings were collected, and NLR and PLR values were calculated. Additionally, routine blood test results and demographic information (sex and age) from 100 healthy controls were included. The healthy control group consisted of individuals who underwent routine physical examinations and had no history of autoimmune diseases or recent infections.

### Statistical analysis

Data were analyzed using SPSS 25.0 (SPSS Inc., Chicago, IL, United States). Normally distributed continuous variables are presented as means ± standard deviations, while non-normally distributed variables are expressed as medians (P25, P75). Intergroup comparisons for normally distributed data were conducted using *t*-tests, whereas Mann–Whitney *U* tests were applied for non-normally distributed data. Categorical data were compared using chi-square tests or Fisher’s exact probability method. Binary logistic regression analysis was performed to evaluate differences in NLR and PLR between NMOSD and MS patients. Receiver operating characteristic (ROC) curve analysis assessed diagnostic accuracy, with areas under the curve (AUC) interpreted as follows: AUC < 0.50 indicates no predictive value, 0.50 ≤ AUC ≤ 0.70 indicates low predictive value, 0.70 < AUC ≤ 0.90 indicates moderate predictive value, and AUC > 0.90 indicates high predictive value. Statistical significance was set at *p* < 0.05.

## Results

A total of 79 patients with NMOSD, 75 patients with MS, and 100 healthy controls were included in this study. The average age of NMOSD patients was 48.19 ± 16.59 years, that of MS patients was 41.79 ± 14.52 years, and that of the healthy control group was 46.21 ± 15.88 years. NMOSD patients were significantly older than MS patients (*p* = 0.012), while no significant differences in age were observed between NMOSD patients and the control group (*p* = 0.418) or between MS patients and the control group (*p* = 0.060). Among NMOSD patients, there were 18 males and 61 females, and among MS patients, there were 23 males and 52 females. No significant differences were found in the male-to-female ratio between the two groups or compared to the control group. The mean EDSS score for NMOSD patients was significantly higher than that for MS patients (*p* = 0.002). Additionally, NMOSD patients exhibited significantly higher EDSS scores at 12 months compared to MS patients (*p* = 0.002) (Table 1).

**Table 1:** Comparison of the serological indicators, clinical, and therapeutic characteristics among NMOSD patients, MS patients and controls.

	NMOSD (*n* = 79)	MS (*n* = 75)	Control (*n* = 100)	*p*-value^a^	*p*-value^b^	*p*-value^c^
Age (years)	48.19 ± 16.59	41.79 ± 14.52	46.21 ± 15.88	0.012	0.418	0.060
Sex (male/female)	18/61	23/52	36/64	0.269	0.056	0.460
White blood cell (×10^9^/L)	7.12 ± 2.30	6.39 ± 1.88	5.77 ± 1.49	0.032	0.000	0.016
Neutrophil (×10^9^/L)	4.83 ± 1.95	3.79 ± 1.68	3.42 ± 1.35	0.001	0.000	0.108
Lymphocyte (×10^9^/L)	1.77 ± 0.61	2.01 ± 0.62	1.88 ± 0.48	0.018	0.197	0.118
Platelet (×10^9^/L)	229.25 ± 65.43	239.07 ± 68.58	222.67 ± 40.60	0.365	0.410	0.068
NLR	3.01 ± 1.62	2.17 ± 1.74	1.98 ± 1.17	0.002	0.000	0.407
PLR	140.66 ± 57.49	130.43 ± 56.74	126.41 ± 40.20	0.269	0.053	0.602
Brain (%)	6 (7.60)	51 (68.00)	—	0.000	—	—
Optic nerve (%)	21 (26.58)	3 (4.00)	—	0.000	—	—
Brainstem/cerebellum (%)	13 (16.46)	19 (25.33)	—	0.175	—	—
Spine (%)	71 (89.87)	60 (80.00)	—	0.086	—	—
EDSS at onset	3 (2, 5)	3 (2, 3)	—	0.002	—	—
EDSS at 1 year later	2 (1, 3)	2 (1, 2)	—	0.002	—	—
Acute phase						
Methylprednisolone (%)	79 (100)	72 (96.00)	—	0.113	—	—
IVIG (%)	0 (0)	1 (1.33)	—	0.487	—	—
PEX (%)	1 (1.3)	0 (0)	—	1.000	—	—
Remission phase						
Teriflunomide (%)	0 (0)	20 (26.67)	—	0.000	—	—
Dimethyl fumarate (%)	0 (0)	1 (1.33)	—	0.487	—	—
Ocrelizumab (%)	0 (0)	5 (6.67)	—	0.026	—	—
MMF (%)	33 (41.78)	8 (10.67)	—	0.000	—	—
Azathioprine (%)	18 (22.78)	5 (6.67)	—	0.005	—	—
Oral prednisone (%)	15 (18.99)	24 (32.00)	—	0.063	—	—
IVIG (%)	11 (13.92)	1 (1.33)	—	0.004		

NLR, neutrophil-to-lymphocyte ratio; PLR, platelet-to-lymphocyte ratio; EDSS, Extended Disability Status Scale; IVIG, intravenous immunoglobulin; PEX, plasma exchange; MMF, mycophenolate mofetil.

a *p*-value: comparison between NMOSD and MS.

b *p*-value: comparison between NMOSD and the controls.

c *p*-value: comparison between MS and the controls.

The number of brain lesions was significantly lower in the NMOSD group than in the MS group, whereas optic nerve involvement was significantly higher in the NMOSD group than in the MS group (both *p* = 0.001). No significant differences were observed in the incidence of brainstem, cerebellar lesions, or spinal cord abnormalities between the two groups (*p* = 0.175, *p* = 0.086).

White blood cell counts were significantly higher in NMOSD patients compared to both MS patients and healthy controls (*p* = 0.032, *p* = 0.001), and white blood cell counts in MS patients were also significantly higher than those in healthy controls (*p* = 0.016). Neutrophil counts were significantly higher in NMOSD patients compared to both MS patients and healthy controls (*p* = 0.001, *p* = 0.001), but no significant difference was observed between MS patients and healthy controls (*p* = 0.108). Lymphocyte counts were significantly lower in NMOSD patients compared to MS patients (*p* = 0.018), with no significant differences compared to healthy controls (*p* = 0.197) or between MS patients and healthy controls (*p* = 0.118). Platelet counts did not differ significantly among the three groups. The NLR was significantly higher in NMOSD patients compared to both MS patients and healthy controls (*p* = 0.002, *p* = 0.001), but no significant difference was observed between MS patients and healthy controls (*p* = 0.407). PLR did not differ significantly among the three groups. After adjusting for age, gender, NLR, and PLR, significant differences were observed in age and NLR between NMOSD and MS patients (*p* = 0.010, *p* = 0.364) (Table 2).

**Table 2:** Logistic regression analysis of the differences in NLR and PLR between NMOSD and MS patients.

	*β*	SE	WALD	*p*	Exp. (*B*)	95% CI for Exp. (*B*)
Sex	−0.373	0.401	0.863	0.353	0.689	0.314–1.512
Age	−0.024	0.011	4.573	0.032	0.976	0.955–0.998
NLR	−0.378	0.146	6.707	0.010	0.685	0.514–0.912
PLR	0.004	0.004	0.824	0.364	1.004	0.996–1.012

NLR, neutrophil-to-lymphocyte ratio; PLR, platelet-to-lymphocyte ratio; EDSS, Extended Disability Status Scale.

Of the NMOSD patients, 59 out of 79 cases (74.7%) tested positive for AQP4 antibodies in blood. The age of AQP4 (+) NMOSD patients was significantly lower than that of AQP4 (−) NMOSD patients (*p* = 0.010). Additionally, the proportion of female patients in the AQP4 (+) NMOSD group was significantly higher compared to the AQP4 (−) NMOSD group (*p* = 0.006). Notably, the blood lymphocyte count in AQP4 (+) NMOSD patients was significantly higher than that in AQP4 (−) patients (*p* = 0.044). However, no significant statistical differences were observed in other blood parameters, including NLR, PLR, and EDSS scores, between the two groups (Table 3).

**Table 3:** Comparison of serological indicators and EDSS scores in NMOSD patients with AQP4 (+) and AQP4 (−) groups.

	AQP4 (+) (*n* = 59)	AQP4 (−) (*n* = 20)	*p*-value
Age (years)	45.91 ± 17.57	54.90 ± 11.11	0.010
Sex (male/female)	9/50	9/11	0.006
White blood cell (×10^9^/L)	7.38 ± 2.33	6.36 ± 2.05	0.084
Neutrophil (×10^9^/L)	5.03 ± 1.95	4.23 ± 1.90	0.114
Lymphocyte (×10^9^/L)	1.83 ± 0.66	1.59 ± 0.34	0.044
Platelet (×10^9^/L)	233.17 ± 66.60	217.70 ± 62.06	0.364
NLR	3.09 ± 1.69	2.78 ± 1.39	0.456
PLR	141.52 ± 63.77	138.05 ± 33.81	0.757
EDSS at onset	4 (2, 5)	3 (2, 4)	0.177
EDSS at 1 year later	2 (1, 3)	2 (1, 2)	0.061

NLR, neutrophil-to-lymphocyte ratio; PLR, platelet-to-lymphocyte ratio; EDSS, Extended Disability Status Scale.

**Figure 1:**
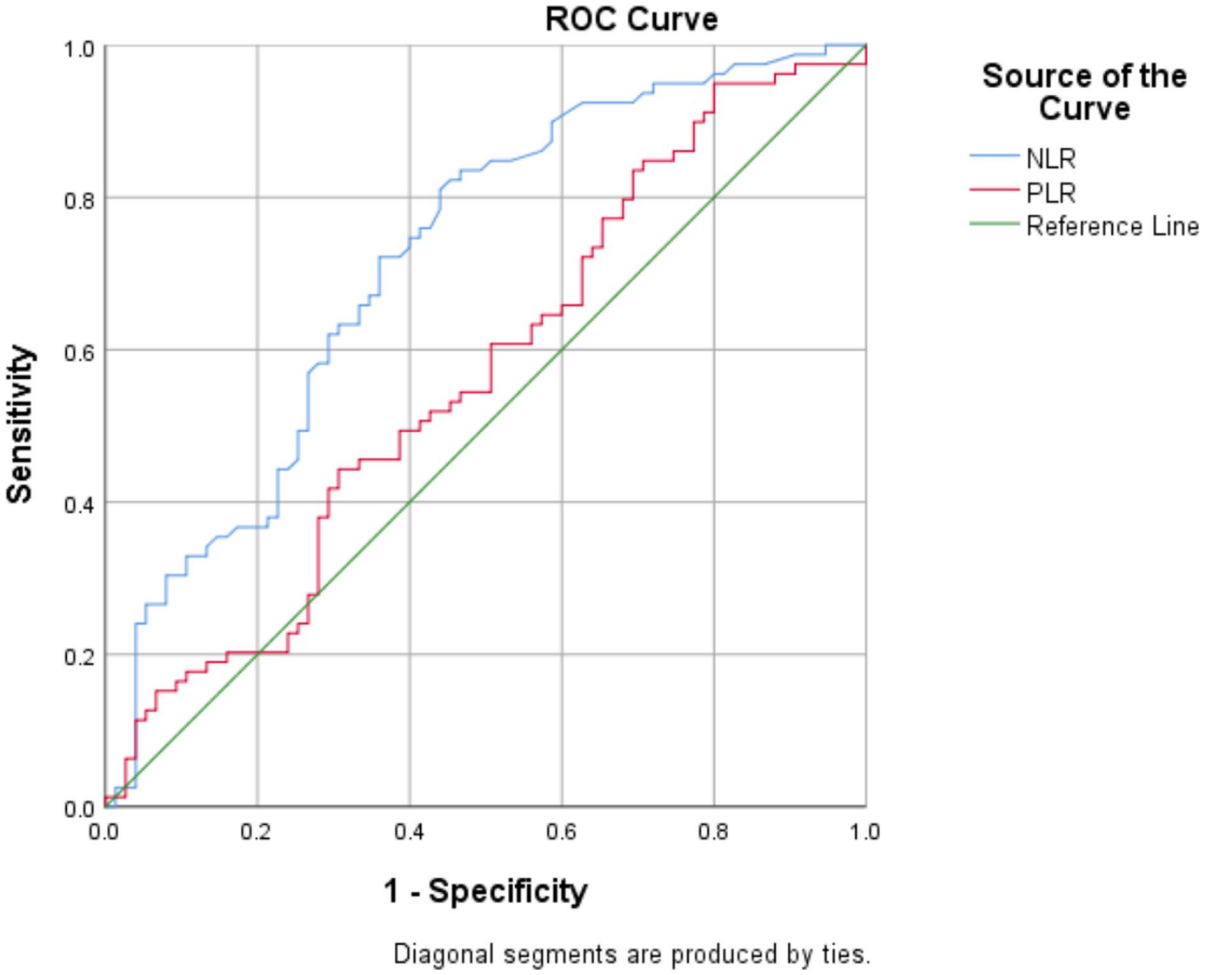
Receiver operating characteristic (ROC) curves of NLR and PLR for the diagnosis patients with NMOSD and patients with MS.

ROC analysis of NLR and PLR for diagnosing NMOSD and MS revealed that the AUC for NLR in NMOSD patients was 0.717 (*p* = 0.001, 95% CI: 0.636 to 0.798), while for PLR it was 0.567 (*p* = 0.152, 95% CI: 0.476 to 0.658). The optimal cutoff value for NLR was determined to be 1.712, yielding a sensitivity of 81.7% and specificity of 55.4%, as shown in Figure 1.

The median NLR in NMOSD patients was 2.44, and these patients were accordingly divided into a low NLR group (NLR < 2.44) and a high NLR group (NLR ≥ 2.44). No significant differences in gender or age were observed between the two groups (*p* = 0.302, *p* = 0.312). The positive rate of serum AQP4 antibodies did not differ significantly between the two groups (*p* = 0.137). White blood cell counts and neutrophil counts were significantly lower in the low NLR group compared to the high NLR group (*p* = 0.001, *p* = 0.001), while lymphocyte counts were higher in the low NLR group (*p* = 0.001). Platelet counts did not differ significantly between the two groups (*p* = 0.360). PLR was significantly lower in the low NLR group compared to the high NLR group (*p* = 0.011). The Pearson correlation coefficient between NLR and PLR was 0.513, with a *p*-value less than 0.001. Baseline and 12-month EDSS scores were significantly lower in the low NLR group compared to the high NLR group (*p* = 0.008, *p* = 0.006) (Table 4).

**Table 4:** Comparison of serological indicators and EDSS scores in NMOSD patients with high and low NLR groups.

	NLR < 2.44	NLR ≥ 2.44	*p*-value
Age (years)	46.28 ± 16.63	50.15 ± 16.53	0.302
Sex (male/female)	11/29	7/32	0.312
AQP4 (−)/(+)	13/27	7/32	0.137
White blood cell (×10^9^/L)	6.28 ± 2.00	7.99 ± 2.27	0.001
Neutrophil (×10^9^/L)	3.69 ± 1.31	5.99 ± 1.82	0.000
Lymphocyte (×10^9^/L)	2.04 ± 0.63	1.50 ± 0.45	0.000
Platelet (×10^9^/L)	235.95 ± 65.83	222.38 ± 65.15	0.360
NLR	1.83 ± 0.40	4.22 ± 1.49	0.000
PLR	124.60 ± 48.62	157.12 ± 61.70	0.011
EDSS at onset (IQR)	3 (2, 4)	4 (3, 5)	0.008
EDSS at 1 year later (IQR)	2 (1, 3)	3 (2, 4)	0.006

NLR, neutrophil-to-lymphocyte ratio; PLR, platelet-to-lymphocyte ratio; EDSS, Extended Disability Status Scale.

In MS patients, the median NLR was 1.68, and they were similarly categorized into low (NLR < 1.68) and high NLR groups (NLR ≥ 1.68). No significant differences in gender or age were observed between the two groups (*p* = 0.398, *p* = 0.170). White blood cell counts and neutrophil counts were significantly lower in the low NLR group compared to the high NLR group (*p* = 0.002, *p* = 0.001), while lymphocyte counts were higher in the low NLR group (*p* = 0.001). Platelet counts did not differ significantly between the two groups (*p* = 0.119). NLR and PLR were significantly lower in the low NLR group compared to the high NLR group (*p* = 0.001, *p* = 0.018). The Pearson correlation coefficient between NLR and PLR was 0.664, with a *p*-value less than 0.001. Baseline and 12-month EDSS scores were significantly lower in the low NLR group compared to the high NLR group (*p* = 0.001, *p* = 0.003) (Table 5).

**Table 5:** Comparison of serological indicators and EDSS scores in MS patients with high and low NLR groups.

	NLR < 1.68	NLR ≥ 1.68	*p*-value
Age (years)	40.45 ± 14.66	43.31 ± 14.40	0.398
Sex (male/female)	15/25	8/27	0.170
White blood cell (×10^9^/L)	5.76 ± 1.29	7.11 ± 2.19	0.002
Neutrophil (×10^9^/L)	2.89 ± 0.80	4.81 ± 1.84	0.000
Lymphocyte (×10^9^/L)	2.29 ± 0.55	1.69 ± 0.53	0.000
Platelet (×10^9^/L)	246.73 ± 68.93	224.19 ± 65.41	0.119
NLR	1.28 ± 0.26	3.19 ± 2.12	0.000
PLR	115.46 ± 40.00	147.55 ± 67.88	0.018
EDSS at onset (IQR)	2 (2, 3)	3 (2, 5)	0.001
EDSS at 1 year later (IQR)	2 (1, 2)	2 (1, 3)	0.003

NLR, neutrophil-to-lymphocyte ratio; PLR, platelet-to-lymphocyte ratio; EDSS, Extended Disability Status Scale.

Among the 79 patients with NMOSD, all received methylprednisolone therapy during the acute phase, and one patient also underwent plasma exchange. Additionally, 33 were treated with mycophenolate mofetil (MMF), 11 with intravenous immunoglobulin (IVIG), 5 with rituximab, 18 with azathioprine, and 15 received oral prednisone therapy alone. Among the 75 patients with MS, 72 received methylprednisolone therapy during the acute phase, while one patient was treated with IVIG. During the remission phase, treatments included oral prednisone for 24 patients, teriflunomide for 20, MMF for 8, dimethyl fumarate for 1, ocrelizumab for 5, azathioprine for 5, and 12 patients received no treatment (Table 1).

## Discussion

We observed that the peripheral blood neutrophil count was significantly higher in NMOSD patients compared to MS patients and healthy controls. Neutrophils, as the most abundant cell type in the innate immune system, are closely associated with the pathogenesis of NMOSD (20, 21). Studies on animal models of experimental autoimmune encephalomyelitis have demonstrated that reducing neutrophil levels may delay disease progression (22). Neutrophils play a critical role in the pathogenesis of experimental autoimmune encephalomyelitis by producing cytokines, damaging the blood–brain barrier, and promoting inflammation in the brain parenchyma (22). It is now well established that neutrophils are the primary inflammatory cells involved in the early stages of NMOSD pathogenesis (23). Inflammatory cells infiltrating NMOSD lesions primarily consist of neutrophils, macrophages, and eosinophils, with relatively few T lymphocytes (20). During NMOSD pathogenesis, neutrophils are the first inflammatory cells to enter the lesion, potentially within a few hours after inflammation onset. Within 12 h of onset, perivascular complement activation, AQP4 loss, and early myelin damage occur. In this study, the WBC counts and absolute neutrophil counts during NMOSD episodes were significantly higher than those in MS patients and the control group, consistent with the pathophysiological process of NMOSD. Unlike MS neutrophils, NMOSD neutrophils exhibit diminished adhesion and migratory capabilities, reduced production of reactive oxygen species, and decreased degranulation (24). Therefore, NMOSD treatment should aim to block persistent inflammation caused by neutrophil apoptosis, promote neutrophil apoptosis, enhance inflammation regression, inhibit excessive tissue injury, and prevent chronic inflammation (25). NMOSD is frequently associated with other autoimmune diseases, such as Sjögren syndrome and myasthenia gravis. To minimize the potential confounding effects of these comorbid conditions and their treatments on the study outcomes, patients with NMOSD accompanied by other autoimmune disorders were excluded from this study.

Additionally, the NLR was elevated in NMOSD patients compared to both MS patients and healthy controls. The NLR serves as a systemic inflammatory marker derived from routine blood tests, which are cost-effective and easily accessible. It reflects both the increase in neutrophils and the decrease in lymphocytes during the inflammatory response. The NLR has been correlated not only with some autoimmune diseases (17, 26) but also with neurological disorders such as Parkinson’s disease (27) and Alzheimer’s disease (28). Several studies have confirmed that the NLR correlates with the prognosis of diseases like rheumatoid arthritis (17), cancer (29, 30), and stroke (31). However, studies on the relationship between NLR and NMOSD remain scarce. Neutrophils are involved in NMOSD pathogenesis (20, 22, 23), and the NLR can reflect changes in neutrophils *in vivo*. Some studies have found that elevated NLR in MS patients is associated with an increased risk of relapse within 2 years (32), exhibits a weak correlation with the MS severity score (33), and serves as an independent predictor of disability progression (7). Conversely, another study reported no association between NLR and EDSS (9), and no significant relationship was found between the annualized relapse rate (ARR) and NLR values (34). Our study revealed that the NLR was significantly higher in NMOSD patients experiencing episodes compared to MS patients and controls. This change was attributed to an increase in the absolute neutrophil count and a decrease in the absolute lymphocyte count. ROC analysis indicated that the NLR holds significant value in distinguishing NMOSD from MS, demonstrating relatively high sensitivity but limited specificity. Further investigations showed that NMOSD patients in the low NLR group (NLR < 2.44) exhibited significantly lower baseline and 12-month EDSS scores compared to those in the high NLR group (NLR ≥ 2.44). Several studies have demonstrated that the NLR is significantly elevated in NMOSD patients, with a moderate to low correlation between elevated NLR and poor prognosis in NMOSD patients (14, 15). Similarly, in MS patients, the low NLR group (NLR < 1.68) showed significantly lower baseline and 12-month EDSS scores compared to the high NLR group (NLR ≥ 1.68), consistent with previous research (7).

PLR is increasingly recognized as an important indicator of inflammation. When combined with NLR, PLR can more accurately reflect *in vivo* inflammatory changes while minimizing the influence of other factors on the absolute counts of leukocyte subtypes. The potential discriminatory and prognostic value of PLR in NMOSD and MS remains controversial. PLR has been shown to effectively distinguish between NMOSD patients in the active phase and those in remission (18). Studies indicate that an increased PLR during NMOSD can predict the severity of neurological disability within 2 years in NMOSD patients (18, 19). In the study by Carnero et al. (19) both NMOSD and MS patients were experiencing their first relapse, with a significant difference in baseline PLR but no significant difference in NLR. In contrast, Li et al. (35) investigated 249 NMOSD patients, 244 MS patients, and 249 healthy controls, and found no significant difference in PLR between NMOSD and MS groups, while NLR was significantly different. Notably, patients in both disease groups in Li’s et al. (35) study were assessed during acute attacks. This study indicates that there is no significant difference in PLR values between NMOSD and MS patients, potentially due to variations in baseline characteristics, inclusion criteria, and medication conditions across studies.

In this study, patients had not used glucocorticoids for one month prior to enrollment and had abstained from immunosuppressants or disease-modifying therapies (DMTs) for at least 3 months before enrollment. These exclusion criteria were implemented to reduce potential confounding effects of medications on hematological parameters. Specifically, glucocorticoid use can alter levels of peripheral blood white cells, neutrophils, and lymphocytes, thereby influencing NLR and PLR values. Similarly, immunosuppressants and DMTs, such as teriflunomide, may affect blood cell counts, necessitating the exclusion of patients who recently used these medications.

This study has several limitations. First, it was conducted as a single-center study with a relatively small sample size, warranting further expansion to include more participants. Second, variations in BMI or weight may affect inflammatory markers such as the NLR, potentially introducing bias into the results (36). This study was neither fully controlled nor matched between groups, which might have led to confounding factors. These issues should be addressed in future research to enhance the validity and robustness of the findings. Thirdly, patients received different medications following relapse, which could impact their prognosis and EDSS scores after 12 months. Lastly, NMOSD patients tend to be older than MS patients, which might introduce age-related biases affecting the study conclusions.

## Conclusion

In summary, our findings demonstrate that NLR values are significantly higher in NMOSD patients compared to MS patients, suggesting its potential utility as an auxiliary tool for differentiating acute attacks or relapses in NMOSD from those in MS. Patients with high NLR values exhibited higher EDSS scores at the one-year follow-up compared to those with lower NLR values. Thus, NLR may serve as a prognostic indicator for NMOSD and MS patients 1 year following an event.

## Data Availability

The raw data supporting the conclusions of this article will be made available by the authors, without undue reservation.

## References

[1] Wingerchuk DM Brenda B Bennelt JL Cabre P Carroll W Chitnis T. International consensus diagnostic criteria for neuromyelitis optica spectrum disorders. Neurology. (2015) 85:177–89. doi: 10.1212/WNL.0000000000001729 26092914 PMC4515040

[2] Thompson AJ Banwell BL Barkhof F Carroll WM Coetzee T Comi G. Diagnosis of multiple sclerosis: 2017 revisions of the McDonald criteria. Lancet Neurol. (2018) 17:162–73. doi: 10.1016/S1474-4422(17)30470-2, PMID: 29275977

[3] Levraut M Landes-Chateau C Mondot L Cohen M Lebrun-Frenay C. The kappa free light chains index and central vein sign: two new biomarkers for multiple sclerosis diagnosis. Neurol Ther. (2025) 14:711–31. doi: 10.1007/s40120-025-00737-7, PMID: 40189723 PMC12089642

[4] Tillack K Naegele M Haueis C Schippling S Wandinger KP Martin R. Gender differences in circulating levels of neutrophil extracellular traps in serum of multiple sclerosis patients. J Neuroimmunol. (2013) 261:108–19. doi: 10.1016/j.jneuroim.2013.05.004, PMID: 23735283

[5] Núñez J Núñez E Bodí V Sanchis J Miñana G Mainar L. Usefulness of the neutrophil to lymphocyte ratio in predicting long-term mortality in ST segment elevation myocardial infarction. Am J Cardiol. (2008) 101:747–52. doi: 10.1016/j.amjcard.2007.11.004, PMID: 18328833

[6] Günay E Sarınç Ulaşlı S Akar O Ahsen A Günay S Koyuncu T. Neutrophil to-lymphocyte ratio in chronic obstructive pulmonary disease: a retrospective study. Inflammation. (2014) 37:374–80. doi: 10.1007/s10753-013-9749-1, PMID: 24078279

[7] Demirci S Demirci S Kutluhan S Koyuncuoglu HR Yurekli VA. The clinical significance of the neutrophil-to-lymphocyte ratio in multiple sclerosis. Int J Neurosci. (2016) 126:700–6. doi: 10.3109/00207454.2015.1050492, PMID: 26000934

[8] D’Amico E Zanghì A Romano A Sciandra M GAM P Patti F. The neutrophil-to-lymphocyte ratio is related to disease activity in relapsing remitting multiple sclerosis. Cells. (2019) 8:1114. doi: 10.3390/cells8101114 31547008 PMC6830321

[9] Bisgaard AK Pihl-Jensen G Frederiksen JL. The neutrophil-to-lymphocyte ratio as disease activity marker in multiple sclerosis and optic neuritis. Mult Scler Relat Disord. (2017) 18:213–7. doi: 10.1016/j.msard.2017.10.009, PMID: 29141813

[10] Lin J Xue B Li J Xu H Huang X Yao Z. Neutrophil to lymphocyte ratio may be a helpful marker to evaluate disease activity in NMOSD. Neurol Sci. (2017) 38:1859–63. doi: 10.1007/s10072-017-3068-5 28779361

[11] Lin L Ji M Wu Y Hang H Lu J. Neutrophil to lymphocyte ratio may be a useful marker in distinguishing MOGAD and MS and platelet to lymphocyte ratio associated with MOGAD activity. Mult Scler Relat Disord. (2023) 71:104570. doi: 10.1016/j.msard.2023.104570, PMID: 36827875

[12] Duan Z Feng J. Comparison of neutrophil-to-lymphocyte ratio between myelin oligodendrocyte glycoprotein antibody-associated disease and aquaporin-4 antibody-positive neuromyelitis optica spectrum disorders in adults. J Clin Neurosci. (2022) 101:89–93. doi: 10.1016/j.jocn.2022.05.002, PMID: 35569419

[13] Zhou Y Xie H Zhao Y Zhang J Li Y Duan R. Neutrophil-to-lymphocyte ratio on admission is an independent risk factor for the severity of neurological impairment at disease onset in patients with a first episode of neuromyelitis optica spectrum disorder. Neuropsychiatr Dis Treat. (2021) 17:1493–503. doi: 10.2147/NDT.S311942, PMID: 34040376 PMC8140946

[14] Cabanillas-Lazo M Cruzalegui-Bazán C Pascual-Guevara M Quispe-Vicuña C Terry-Escalante FA Mori N. Clinical and imagenologic significance of the neutrophil-to-lymphocyte ratio in neuromyelitis optica spectrum disorder: a systematic review with meta-analysis. PLoS One. (2023) 18:e0281064. doi: 10.1371/journal.pone.0281064, PMID: 36758016 PMC9910629

[15] Devlin L Gombolay G. The neutrophil-to-lymphocyte ratio and the monocyte-to-lymphocyte ratio predict expanded disability status scale score at one year in pediatric neuromyelitis optica spectrum disorder but not in multiple sclerosis. Pediatr Neurol. (2023) 143:84–8. doi: 10.1016/j.pediatrneurol.2023.03.009, PMID: 37044044 PMC10205676

[16] Qin B Ma N Tang Q Wei T Yang M Fu H. Neutrophil to lymphocyte ratio (NLR) and platelet to lymphocyte ratio (PLR) were useful markers in assessment of inflammatory response and disease activity in SLE patients. Mod Rheumatol. (2016) 26:372–6. doi: 10.3109/14397595.2015.1091136, PMID: 26403379

[17] Fu H Qin B Hu Z Ma N Yang M Wei T. Neutrophil-and platelet-to-lymphocyte ratios are correlated with disease activity in rheumatoid arthritis. Clin Lab. (2015) 61:269–73. doi: 10.7754/clin.lab.2014.140927 25974992

[18] Fang X Sun S Yang T Liu X. Predictive role of blood-based indicators in neuromyelitis optica spectrum disorders. Front Neurosci. (2023) 17:1097490. doi: 10.3389/fnins.2023.1097490, PMID: 37090792 PMC10115963

[19] Carnero Contentti E López PA Criniti J Pettinicchi JP Cristiano E Patrucco L. Platelet-to-lymphocyte ratio differs between MS and NMOSD at disease onset and predict disability. Mult Scler Relat Disord. (2022) 58:103507. doi: 10.1016/j.msard.2022.103507, PMID: 35030372

[20] Saadoun S Waters P MacDonald C Bell BA Vincent A Verkman AS. Neutrophil protease inhibition reduces neuromyelitis optica-immunoglobulin G-induced damage in mouse brain. Ann Neurol. (2012) 71:323–33. doi: 10.1002/ana.22686, PMID: 22374891 PMC3643520

[21] Zhang H Verkman AS. Eosinophil pathogenicity mechanisms and therapeutics in neuromyelitis optica. J Clin Invest. (2013) 123:2306–16. doi: 10.1172/JCI67554 23563310 PMC3635742

[22] Pierson ER Wagner CA Goverman JM. The contribution of neutrophils to CNS autoimmunity. Clin Immunol. (2018) 189:23–8. doi: 10.1016/j.clim.2016.06.017, PMID: 27377536 PMC5203971

[23] Gong Y Zhang YL Wang Z Song HH Liu YC Lv AW. Tanshinone IIA alleviates brain damage in a mouse model of neuromyelitis optica spectrum disorder by inducing neutrophil apoptosis. J Neuroinflammation. (2020) 17:198. doi: 10.1186/s12974-020-01874-6 32586353 PMC7318433

[24] Hertwig L Pache F Romero-Suarez S Stürner KH Borisow N Behrens J. Distinct functionality of neutrophils in multiple sclerosis and neuromyelitis optica. Mult Scler. (2016) 22:160–73. doi: 10.1177/1352458515586084, PMID: 26540731

[25] Gilroy DW Lawrence T Perretti M Rossi AG. Inflammatory resolution: new opportunities for drug discovery. Nat Rev Drug Discov. (2004) 3:401–16. doi: 10.1038/nrd1383 15136788

[26] Acarturk G Acay A Demir K Ulu MS Ahsen A Yuksel S. Neutrophil-to-lymphocyte ratio in inflammatory bowel disease-as a new predictor of disease severity. Bratisl Lek Listy. (2015) 16:213–7. doi: 10.4149/bll_2015_041 25773946

[27] Akil E Bulut A Kaplan I Özdemir HH Arslan D Aluçlu MU. The increase of carcinoembryonic antigen (CEA), high-sensitivity C-reactive protein, and neutrophil/lymphocyte ratio in Parkinson’s disease. Neurol Sci. (2015) 36:423–8. doi: 10.1007/s10072-014-1976-1, PMID: 25288159

[28] Kuyumcu ME Yesil Y Oztürk ZA Kizilarslanoğlu C Etgül S Halil M. The evaluation of neutrophil-lymphocyte ratio in Alzheimer’s disease. Dement Geriatr Cogn Disord. (2012) 34:69–74. doi: 10.1159/000341583, PMID: 22922667

[29] Sun Y Zhang L. The clinical use of pretreatment NLR, PLR, and LMR in patients with esophageal squamous cell carcinoma: evidence from a meta-analysis. Cancer Manag Res. (2018) 22:6167–79. doi: 10.2147/CMAR.S171035 PMC6257133 30538564

[30] Wei B Yao M Xing C Wang W Yao J Hong Y. The neutrophil lymphocyte ratio is associated with breast cancer prognosis: an updated systematic review and meta-analysis. Onco Targets Ther. (2016) 9:5567–75. doi: 10.2147/OTT.S108419 27660475 PMC5021064

[31] Tokgoz S Kayrak M Akpinar Z Seyithanoğlu A Güney F Yürüten B. Neutrophil lymphocyte ratio as a predictor of stroke. J Stroke Cerebrovasc Dis. (2013) 22:1169–74. doi: 10.1016/j.jstrokecerebrovasdis.2013.01.011, PMID: 23498372

[32] Huang WC Lin HC Yang YH Hsu CW Chen NC Tsai WC. Neutrophil-to-lymphocyte ratio and monocyte-to-lymphocyte ratio are associated with a 2-year relapse in patients with multiple sclerosis. Mult Scler Relat Disord. (2022) 58:103514. doi: 10.1016/j.msard.2022.103514 35032880

[33] Hasselbalch IC Søndergaard HB Koch-Henriksen N Olsson A Ullum H Sellebjerg F. The neutrophil-to-lymphocyte ratio is associated with multiple sclerosis. Mult Scler J Exp Transl Clin. (2018) 4:2055217318813183. doi: 10.1177/2055217318813183 30515298 PMC6262498

[34] Kostic M Dzopalic T Zivanovic S Zivkovic N Cvetanovic A Stojanovic I. IL-17 and glutamate excitotoxicity in the pathogenesis of multiple sclerosis. Scand J Immunol. (2014) 79:181–6. doi: 10.1111/sji.12147, PMID: 24383677

[35] Li X Jiang W Li G Ding Y Li H Sun J. Inflammatory and nutritional markers as indicators for diagnosing and assessing disease activity in MS and NMOSD. J Inflamm Res. (2024) 17:10065–78. doi: 10.2147/JIR.S489502 39628706 PMC11613729

[36] Murad LD Silva TQ Schilithz AOC Monteiro MC Murad LB Fialho E. Body mass index alters the predictive value of the neutrophil-to-lymphocyte ratio and systemic inflammation response index in laryngeal squamous cell carcinoma patients. Nutr Cancer. (2022) 74:1261–9. doi: 10.1080/01635581.2021.1952447, PMID: 34278900

